# Association between Khorana score and prognosis in cancer patients with stage IV treated with immune checkpoint inhibitors ∼ factor analysis in Khorana score related to overall survival

**DOI:** 10.3389/fimmu.2025.1633398

**Published:** 2025-09-29

**Authors:** Takayuki Ide, Taisuke Araki, Tomonobu Koizumi

**Affiliations:** ^1^ Department of Pharmacy, Shinshu University Hospital, Nagano, Japan; ^2^ First Department of Internal Medicine, Shinshu University School of Medicine, Nagano, Japan; ^3^ Shinshu Cancer Center, Shinshu University Hospital, Nagano, Japan

**Keywords:** Khorana score, prognosis, immune checkpoint inhibitor, risk factor, patient with cancer

## Abstract

**Purpose:**

The Khorana venous thromboembolism risk assessment score (Khorana score) is an established tool for risk stratification of thromboembolism in patients with cancer. There have been few reports on the relation between Khorana score and prognosis in patients after treatment with immune checkpoint inhibitors (ICIs). The present study was performed to evaluate the association between prognosis and Khorana score in patients with stage IV cancer treated with ICIs.

**Methods:**

We conducted a retrospective chart survey of patients receiving at least one ICI at Shinshu University Hospital between September 2014 and October 2021. Age, sex, cancer type, body mass index, laboratory data at commencement of treatment, and patient outcomes were obtained from electronic medical records. Khorana score was calculated based on cancer type and biomarkers.

**Results:**

The study population consisted of 407 patients (71.0% men) with a median age of 70.0 years (interquartile range [IQR], 63.0–76.0) and a median follow-up of 15.1 months (range, 0.16-72.0). Nivolumab was the most commonly used ICI (60.4%). The median survival time (MST) for all patients was 17.5 months (95% CI, 14.4-20.8). There were significant differences in MST between the low-risk, intermediate-risk and high-risk groups according to Khorana score (*p* < 0.001, p = 0.022, respectively). With regard to each component of the Khorana score, exploratory univariate analysis of risk factors revealed significant differences in white blood cell (WBC) count, hemoglobin (Hb) level, and some cancer types (*p* = 0.009, *p* < 0.001, and *p* = 0.006, respectively). Hb level < 10 g/dL was identified as a risk factor on the Cox proportional hazards regression analysis (Hazard Ratio, 1.78; 95% CI, 1.21-2.60; *p* = 0.003).

**Conclusion:**

Our results suggested that Khorana score at the start of ICIs treatment was related to prognosis of patients with stage IV cancer. In particular, Hb level < 10 g/dL before commencement of treatment was shown to be an independent risk factor affecting prognosis.

## Introduction

Venous thromboembolism (VTE) is a typical complication seen in cancer patients (1). The Khorana VTE risk assessment score (Khorana score) (2) is an established tool for risk stratification of thromboembolism in patients with cancer, which has been adopted by National Comprehensive Cancer Network (3), American Society of Clinical Oncology (4), and European Society for Medical Oncology (5) guidelines.

Immune checkpoint inhibitors (ICIs) have significantly improved clinical outcomes of patients with various malignancies (6–10). Long-term follow-up of patients in clinical trials of the anti-PD-1 inhibitor nivolumab demonstrated an overall survival (OS) plateau with a long tail on the survival curve (11). On the other hand, patients with advanced non-small cell lung cancer (NSCLC) and poor performance status (PS) were found to have significantly shorter survival after treatment with ICIs compared to those with favorable PS (12).

There have been few reports regarding the relation between Khorana score and prognosis in patients after treatment with ICIs (13–16). In addition, the Khorana score is composed of cancer type, white blood cell (WBC) count, hemoglobin (Hb) level, platelet (Plt) count, and body mass index (BMI), but it is not clear which of these factors are most closely associated with prognosis.

The present study was performed to evaluate the association between prognosis and Khorana score in patients with stage IV cancer treated with ICIs.

## Methods

### Subjects

**Figure 1:**
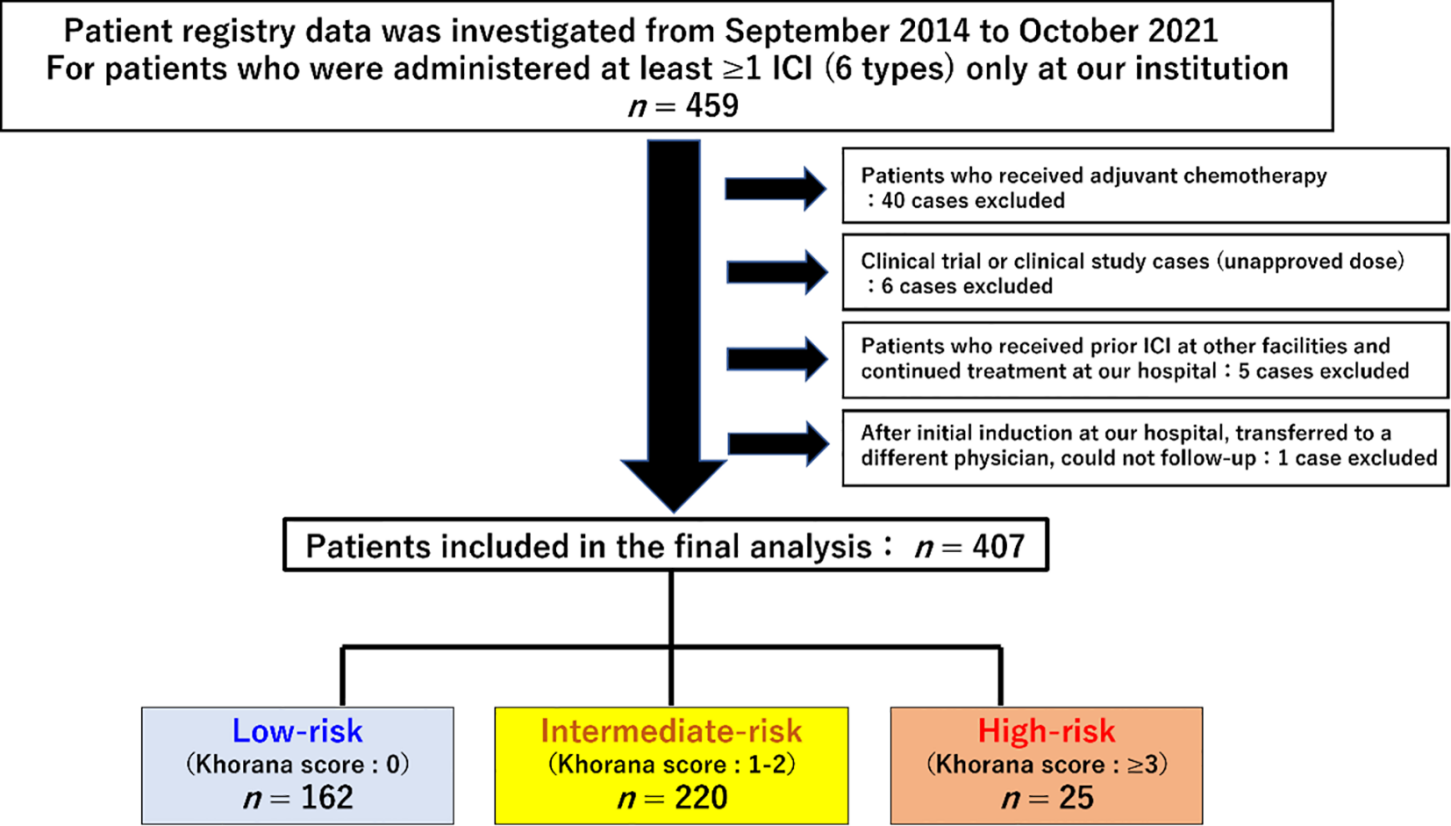
Flow diagram and clinical characteristics of the study population. A total of 459 patients were initially selected, 52 of whom were excluded, and finally 407 patients were included in the analysis.

Patients treated with ICIs at Shinshu University Hospital between September 1, 2014, and October 31, 2021, were retrospectively enrolled in this study. To ensure a minimum observation period of 6 months, the data cutoff was set as June 30, 2022. The list of patients was extracted from the medication history database of our hospital pharmacy department. The eligibility criterion was at least one cycle of ICI treatment at our institution, including monotherapy as well as ICI combination therapy. Patients who received cytotoxic anticancer drugs or molecular targeting agents in combination with ICIs were excluded. Baseline clinical information and laboratory data were defined as those determined within 2 weeks prior to commencement of ICI treatment. A total of 407 patients met these criteria and were included in the analysis (**Figure 1**).

### Investigations

All baseline and subsequent clinical data in this retrospective study were extracted from electronic medical records.

### Patient background data

Data regarding age, sex, BMI, cancer type, treatment line, contents of treatment regimen, and use of erythropoiesis stimulating agents were recorded at commencement of ICI treatment.

### Clinical laboratory test values

Blood biomarkers (i.e., WBC count, Hb, and Plt count), were examined retrospectively based on the electronic medical records. The results of the most recent laboratory blood examination prior to ICI administration were used as baseline data.

### Calculation of Khorana score

The Khorana score was calculated based on cancer type: 2 points were added for gastric and pancreatic cancer (cancer types 1), while 1 point was added for lung cancer, gynecological cancer, urothelial cancer, and lymphoma (cancer types 2), and biomarkers, such as WBC count, Plt count, Hb level, or use of erythropoiesis-stimulating agents, and BMI scored 1 point each. A score of 0 indicates low risk, 1–2 indicates intermediate risk, and ≥ 3 was classified as high risk (2).

### Calculation of overall survival

OS was defined as the time from the date of initiation of ICI therapy to the date of death from any cause. Patients alive at the cutoff date were censored at the date of the last known survival. For patients who were untraceable, the cutoff date was the last date of confirmed survival before the cutoff date.

### Statistical analysis

The Kruskal-Wallis test was used for comparison of continuous variables (quantitative data) that did not follow a normal distribution. For comparisons of categorical variables (qualitative data), Fisher’s exact test was used. *Post hoc* tests (Bonferroni’s correction) were performed when significant differences were detected. These univariate analyses were performed as exploratory analyses. For variables where a significant difference was detected, risk factor estimation and hazard ratio calculation were performed using Cox proportional hazards regression analysis. OS was evaluated using the Kaplan-Meier method and comparisons were performed using the log-rank test.

Statistical analyses were performed using EZR (17) Ver. 1.55 (Saitama Medical Center, Jichi Medical University, Japan). In all analyses, *p* < 0.05 (two-tailed) was taken to indicate statistical significance.

## Results

### Subjects

In total, 407 patients were eligible and analyzed, as shown in **Figure 1**. No patients were treated with erythropoiesis-stimulating drugs. Patient background.

Baseline characteristics are summarized in **Table 1**. Briefly, the median age was 70 years, and 71% of patients were men. The most common type of cancer was NSCLC, followed by malignant melanoma, head and neck cancers, and urothelial cancers. Most patients received monotherapy, with nivolumab and pembrolizumab being the predominant regimens.

**Table 1:** Patient characteristics (*n*= 407).

Variable	Median	[IQR]	<Min-Max>
Age at start of ICIs	70.0	[63.0-76.0]	<19.0-89.0>
BMI	21.8	[19.5-24.2]	<13.3-38.1>
	*n*	(%)	(% missing)
Sex			(0)
Male	289	71.0	
Female	118	29.0	
Khorana score at start of ICIs			(0)
0	162	39.8	
1	158	38.8	
2	62	15.2	
3	19	4.7	
4	5	1.2	
5	1	0.2	
Cancer types			(0)
Non-small cell lung cancer	131	32.2	
Malignant melanoma	87	21.4	
Head and neck cancers	62	15.2	
Urothelial cancers	44	10.8	
Renal cell cancer	27	6.6	
Esophageal cancer	17	4.2	
Gastric cancer	14	3.4	
Malignant pleural mesothelioma	9	2.2	
Gynecologic cancers	5	1.2	
Other cancers ^a^	11	2.7	
Therapeutic management			(0)
Monotherapy			
Nivolumab	246	60.4	
Pembrolizumab	113	27.8	
Atezolizumab	14	3.4	
Avelumab	5	1.2	
Ipilimumab	1	0.2	
Combination therapy			
Nivolumab + ipilimumab	28	6.9	
Therapeutic line of ICIs			(0)
1^st^	120	29.5	
2^nd^	172	42.3	
3^rd^	67	16.5	
4^th^	25	6.1	
≥5^th^ (Max:15^th^)	23	5.7	

IQR, interquartile range; Min, minimum; Max, maximum; ICIs, immune checkpoint inhibitors; BMI, body mass index.

a Hodgkin lymphoma (*n* = 2), Pancreatic cancer (*n* = 2), Thymic cancer (*n* = 2), Merkel cell cancer (*n* = 2), Cancer of unknown primary (*n* = 1), Cholangiocellular cancer (*n* = 1), Breast cancer (*n* = 1).

### Number of cases per risk factor of Khorana score

The numbers of cases of each cancer type classified by the Khorana score were as follows: 16 patients had gastric or pancreatic cancer (cancer types 1); 179 patients had lung cancer, malignant lymphoma, gynecological cancers, bladder cancer, or testicular cancers (cancer types 2); and the remaining 212 patients had other cancer types.

**Figure 2:**
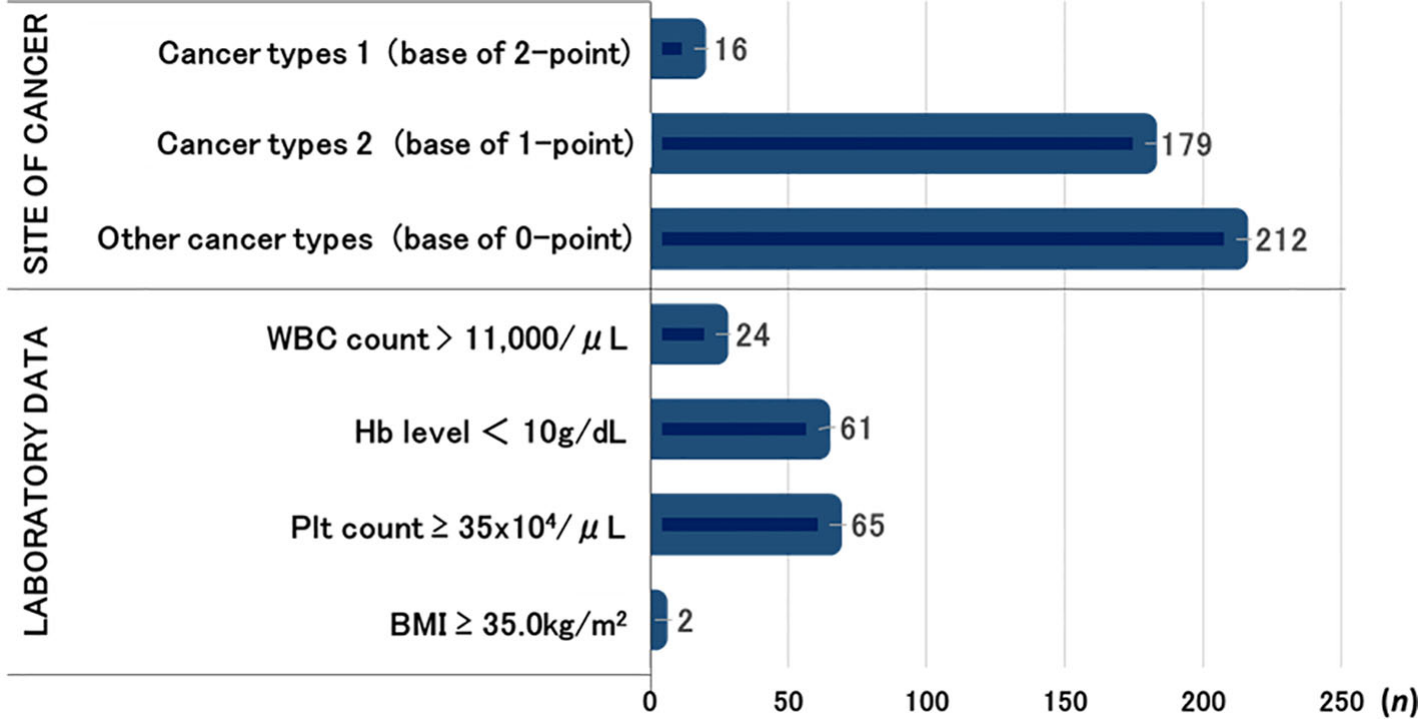
Number of patients by each component of the Khorana score.

The laboratory-based risk factors included WBC count > 11,000/μL (*n* = 24), Hb level < 10 g/dL (*n* = 61), Plt count ≥ 35 × 10^4^/μL (*n* = 65), and BMI ≥ 35 kg/m^2^ (*n* = 2) (**Figure 2**).

### Univariate analysis of patient background by Khorana score risk category

No significant differences were detected in age, sex, or BMI between Khorana score risk categories on univariate analysis. In contrast, laboratory analysis revealed significant differences between risk groups in WBC count, Hb level, and Plt count (*p* < 0.001). Within the WBC fraction, significant differences were also observed in neutrophil, monocyte, and eosinophil counts between groups. There was a significant difference between the first-line and second-line or later treatment lines (*p* = 0.007). No difference was observed between programmed cell death 1 inhibitors and programmed death-ligand 1 inhibitors, nor between ICI monotherapy and combination therapy (**Table 2**).

**Table 2:** Univariative analysis of patient backgrounds.

Variable	Category	Low risk group		Intermediate risk group		High risk group		*p*
		(*n* = 162)		(*n* = 220)		(*n* = 25)		
		Median	IQR	Median	IQR	Median	IQR	
Age		70.0	63.0-76.8	70.0	64.0-75.3	67.0	59.0-71.0	0.281
Sex ( *n* [%] )	Female	51	[31.5]	60	[27.3]	7	[28.0]	0.665
BMI		22.1	19.8-24.6	21.6	19.4-23.9	21.0	19.6-22.9	0.103
WBC		5.28	4.34-6.38	6.44	5.05-8.24	11.87	7.48-13.43	<0.001*
Nut		3.57	2.56-4.51	4.35	3.30-6.08	7.93	5.33-10.79	<0.001*
Lym		1.14	0.79-1.56	1.19	0.85-1.56	1.20	0.97-1.91	0.296
Mon		0.37	0.29-0.45	0.42	0.31-0.54	0.72	0.51-0.93	<0.001*
Eos		0.11	0.05-0.19	0.11	0.06-0.20	0.20	0.09-0.35	0.043*
Hb		12.9	11.6-13.9	11.8	10.4-12.8	9.6	8.1-11.3	<0.001*
Plt		20.7	17.5-25.3	25.1	19.8-32.4	42.3	39.0-51.9	<0.001*
		n						
Therapeutic line 1st vs. > 2nd	1st	63		51		6		0.007*
PD-1 inhibitors vs. PD-L1 inhibitors	PD-1 inhibitors	141		196		23		0.249
ICI single vs. ICI combination	ICI single	145		210		24		0.064

IQR, interquartile range; BMI, body mass index; WBC, white blood cell; Nut, neutrophil; Lym, lymphocyte; Mon, monocyte; Eos, eosinophil; Hb, haemoglobin; Plt, platelet; PD-1, programmed Cell Death 1; PD-L1, programmed Death-Ligand 1; ICI, immune checkpoint inhibitor.

*p < 0.05.

### Overall survival for all patients by Khorana score

**Figure 3:**
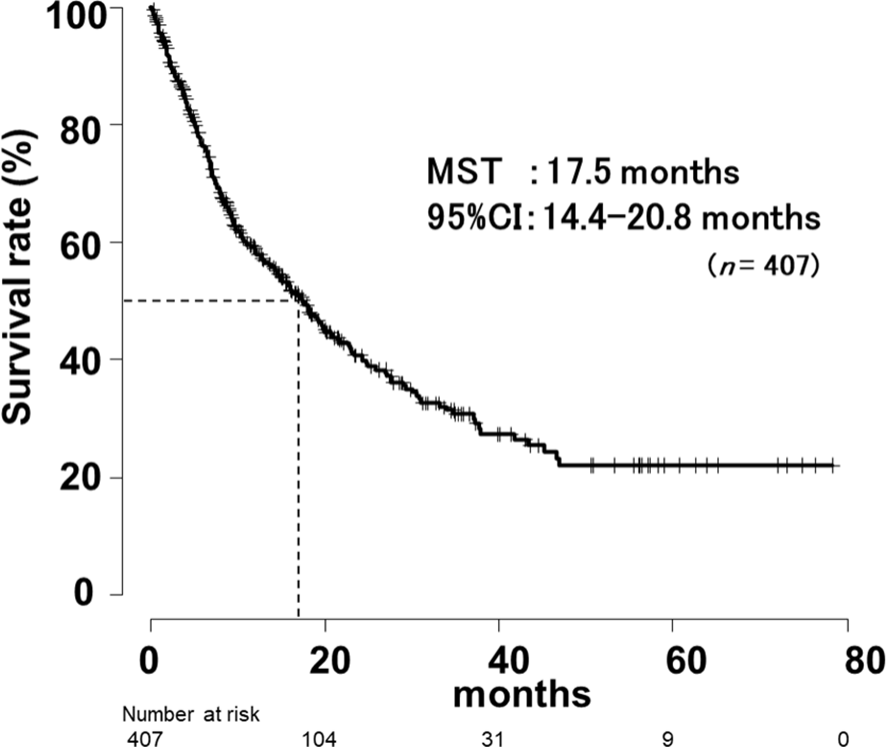
Overall survival curves of all patients in the registered.

In this study, the mean follow-up period was 15.1 months (range, 0.16-72.0 months) after the first dose of ICI. OS for all patients is shown in **Figure 3**. The median survival time (MST) was 17.5 months (95% CI, 14.4-20.8 months).

**Figure 4:**
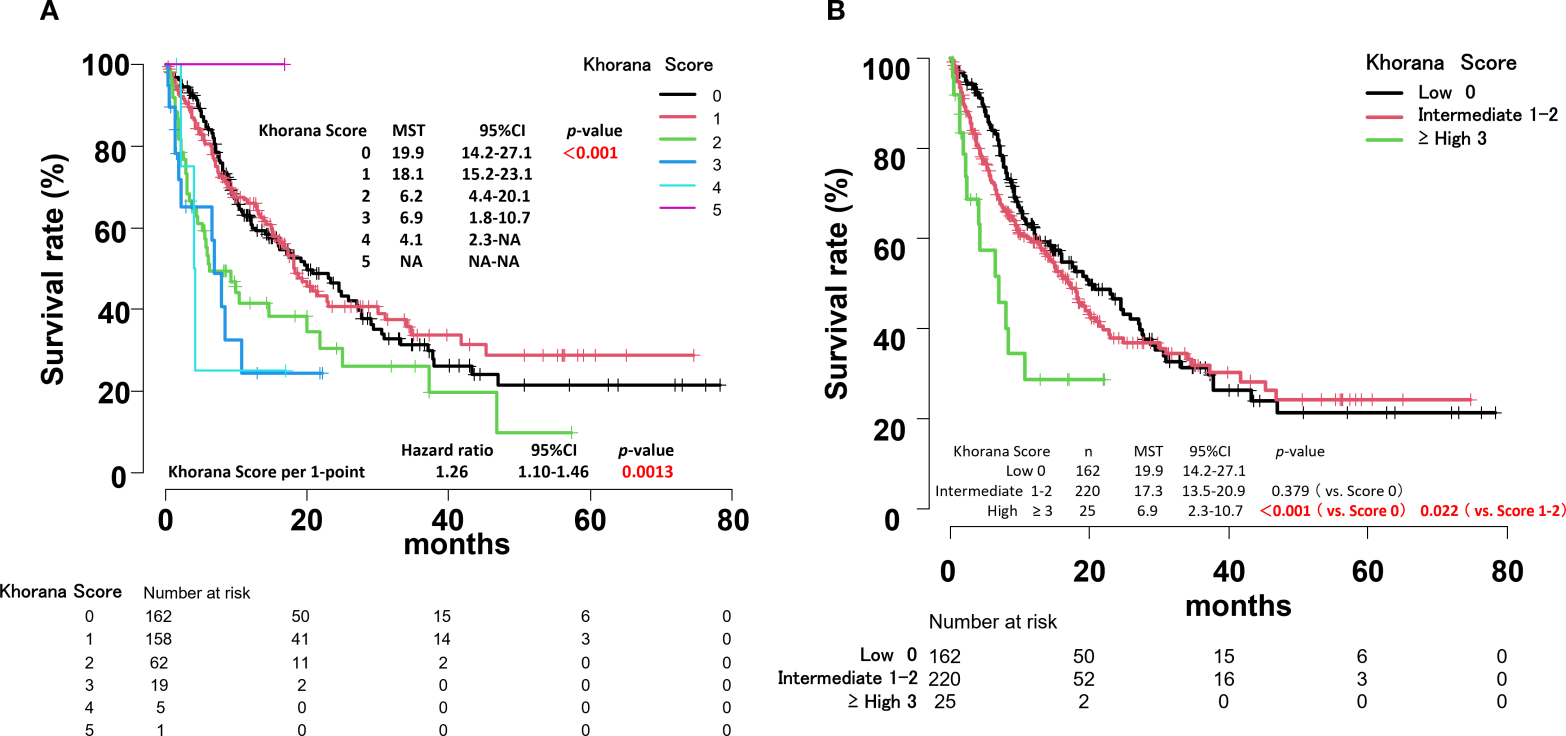
Overall survival curves by Khorana score for all patients **(A)**. Overall survival curves by risk of thrombosis according to Khorana score for all patients **(B)**.

OS by Khorana score can be summarized as follows (**Figure 4A**):

Score 0: MST, 19.9 months (95% CI, 14.2-27.1)
Score 1: MST, 18.1 months (95% CI, 15.2-23.1)
Score 2: MST, 6.2 months (95% CI, 4.4-20.1)
Score 3: MST, 6.9 months (95% CI, 1.8-10.7)
Score 4: MST, 4.1 months (95% CI, 2.3-Not Applicable)
Score 5: only 1 case.

The hazard ratio (HR) increased by 1.26 times for each 1-point increase in the Khorana score (*p* = 0.0013).

### Overall survival by Khorana VTE risk assessment score

There was no significant difference in MST between the low-risk and intermediate-risk groups based on the Khorana VTE risk assessment score. However, a significant difference was observed between the low-risk, intermediate-risk and high-risk groups (*p* < 0.001, *p* = 0.022, respectively) (**Figure 4B**). Within the intermediate-risk group, a comparison between Khorana score 1 and 2 groups revealed a significant difference in MST (*p* = 0.003) (**Figure 4A**, **Supplementary Figure S1**).

After classifying cancer types according to components of the Khorana score (0, 1, and 2 points), comparisons were made by adding 1 point for other risk factors. Significant differences were observed between cancer types 2 and other cancer types (**Supplementary Figure S2**).

### Overall survival for each component of the Khorana score

**Figure 5:**
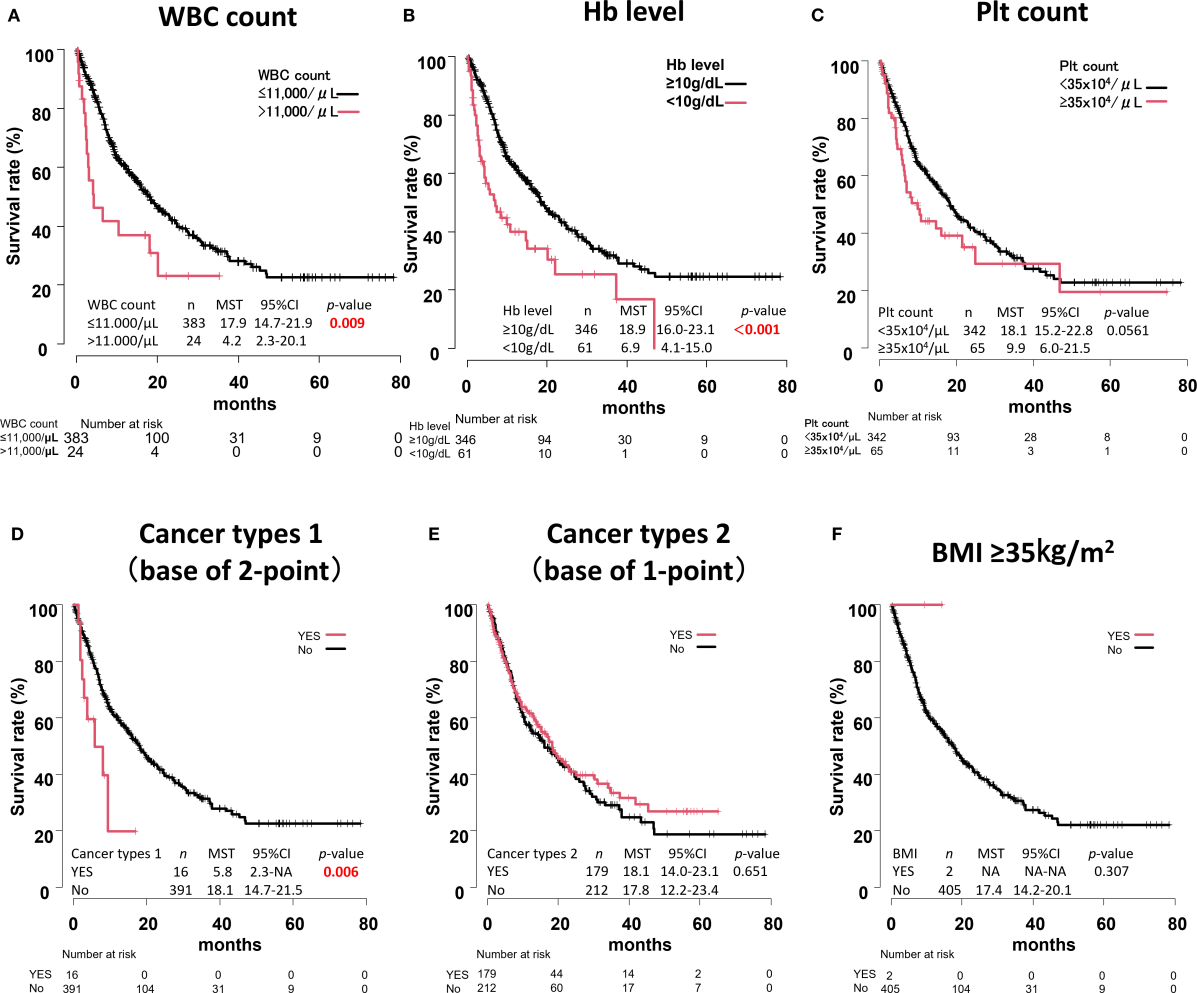
Overall survival curves for each component of the Khorana score. WBC count **(A)**, Hb level **(B)**, Plt count **(C)**, Cancer types 1 **(D)**, Cancer types 2 **(E)**, BMI ≥35kg/m^2^ **(F)**.

OS was then calculated for each component of the Khorana score. Significant differences were found in WBC count, Hb level, and cancer types 1 (*p* = 0.009, *p* < 0.001, and *p* = 0.006, respectively) (**Figures 5A–F**).

### Cox proportional hazards regression analysis of each Khorana score component

Cox proportional hazards regression analysis was performed using each of the 6 components of the Khorana score (WBC count, Hb level, Plt count, cancer types 1, cancer types 2, and BMI) as variables. The results identified Hb level < 10 g/dL (HR, 1.78; 95% CI, 1.21-2.60; *p* = 0.003) as a risk factor (**Table 3**).

**Table 3:** Cox regression analysis for associations between component of Khorana Score, Therapeutic line and OS.

Risk factor	Category	HR	(95% CI)	*p*
WBC count >11,000/μL	Yes	1.54	(0.87-2.72)	0.138
Hb level <10g/dL	Yes	1.78	(1.21-2.60)	0.003*
Plt count >350,000/μL	Yes	1.01	(0.67-1.54)	0.932
BMI ≥ 35.0kg/m^2^	Yes	(-)	(-)	0.994
Cancer types 1 ^a^	Yes	1.36	(0.95-1.95)	0.095
Cancer types 2 ^b^	Yes	0.91	(0.69-1.20)	0.490
Therapeutic line 1st vs. > 2nd	1st	1.20	(0.89-1.61)	0.243

*HR* hazard ratio, *CI* confidence interval, *WBC* white blood cell, *Hb* hemoglobin, *Plt* platelet, *BMI* body mass index.

* *p* < 0.05.

a Gastric cancer or Pancreatic cancer.

b Lung cancers or Gynecologic cancers or Urothelial cancers or Lymphoma.

## Discussion

The MST for all patients in this study was 17.5 months, which was comparable to the median OS of 14.5 months reported previously for patients with NSCLC with PS 0–1 receiving ICI alone (12). MST tended to decrease with each 1-point increase in Khorana score, and there was a significant difference between the high-risk and low-risk groups.

The “cancer types 1” category (gastric, pancreatic cancer), getting 2 points in Khorana score, may contribute to the difference in risk between high *vs*. intermediate and high *vs*. low risk due to its high Khorana score. However, in our study, Hb level < 10 g/dL emerged as an independent prognostic factor in Cox regression analysis.

Previous reports indicate that while the Khorana score risk classification shows no significant difference in OS for metastatic gastric cancer (18), an increased score correlates with poor prognosis in metastatic pancreatic cancer (19). Thus, the association between the score and survival is not evenly distributed across cancer types. When analyzing all cancer types treated with ICIs collectively, as in this study, the influence of specific cancers may be offset. However, this study remains significant in demonstrating trends common to multiple cancers, despite acknowledging these limitations. Future studies with larger sample sizes, examining cancer-specific and organ-specific analyses, are expected to clarify more precise clinical significance.

In the present study, only 25 patients were classified into the high-risk group (Score 3: *n* = 19, Score 4: *n* = 5, Score 5: *n* = 1). The distribution of treatment lines was heterogeneous (1st line *n* = 6, 2nd line *n* = 7, 3rd line *n* = 8, 4th line *n* = 2, 7th and 10th line *n* = 1 each), and cancer types were also imbalanced (NSCLC *n* = 13, gastric cancer *n* = 4, pancreatic cancer *n* = 1, urothelial cancers *n* = 3, others *n* = 4). Given this small number and heterogeneity, the extremely poor prognosis observed in the high-risk group should be interpreted with caution. It is plausible that both the advanced treatment lines and the predominance of cancer types with intrinsically poor outcomes contributed to the results. Nevertheless, our findings underscore that the Khorana score may capture clinically meaningful risk even across diverse cancer types, which warrants further validation in larger, cancer-specific cohorts.

In general, low albumin (20), high lactate dehydrogenase (21, 22), and high C-reactive protein (23) levels before commencement of treatment have been reported to be poor prognostic factors in patients with cancer. In addition, Hb levels below the normal range have been shown to be associated with poor prognosis in metastatic renal cell cancer (24). Furthermore, anemia has been shown to be a prognostic factor in head and neck cancer (25).

On the other hand, in patients with stage IV NSCLC, cancer cachexia—a cancer-associated wasting syndrome characterized by elevated inflammatory markers (CRP > 0.5 mg/dL, interleukin [IL]-6 > 4.0 pg/mL), low Hb (< 12.0 g/dL), and low albumin (< 3.2 g/dL)—has been shown to attenuate the therapeutic efficacy of single-agent ICI treatment (26, 27).

Gou et al. (28) reported that pretreatment Hb levels are associated with progression-free survival (PFS) and OS in immunotherapy for stage IV gastric cancer. Low Hb level (i.e., anemia) is common in patients with cancer and is thought to contribute to intratumoral hypoxia (29), which increases cancer growth and progression and decreases sensitivity to anticancer therapy (30, 31). Zhao L et al. (32) reported that anemia was also associated with T-cell deficiency in a mouse model. It is well known that T cells play important roles in the cancer microenvironment and anticancer responses (33) during ICI treatment. Therefore, anemia may result in a decrease in peritumoral T cell number.

These findings suggest that immunotherapy may be less effective in patients with low Hb levels, which may in turn affect prognosis. Therefore, improving anemia in patients receiving immunotherapy could have a beneficial effect on survival.

### Limitations

There are several limitations in this study. First, this study is a single-center retrospective study, which may introduce selection bias and potentially affect the accuracy and reliability of the results. Second, while this study primarily focuses on the association between the Khorana score and prognosis, it provides limited detail regarding specific aspects of the ICI treatment process (e.g., dose adjustments, reasons for treatment interruption or change, occurrence and management of immune-related adverse events). These factors may influence patient prognosis, and the lack of detailed analysis in these areas may limit the comprehensiveness of the interpretation of the study results. Third, for treatment lines initiated with ICIs, univariate analysis revealed a significant difference between the first-line group and the second-line or later groups (p=0.007). Low-risk patients tended to receive ICI treatment in the first-line or second-line (relatively early treatment lines). It cannot be definitively concluded that this did not affect overall survival (OS). Finally, we inferred that low Hb levels may influence treatment prognosis with immunotherapy. Low Hb is generally recognized as a poor prognostic factor for cancer. Whether it constitutes a prognosis factor specific to immunotherapy remains unclear; further research and discussion are needed.

## Conclusion

This study suggested that Khorana score prior to initiation of ICI treatment may be associated with prognosis in patients with stage IV cancer. An additive evaluation of the individual components of the Khorana score revealed significant prognostic impact. In particular, Hb level < 10 g/dL before commencement of treatment was identified as an independent risk factor associated with poor prognosis.

## Data Availability

The raw data supporting the conclusions of this article will be made available by the authors, without undue reservation.

## Supplementary material

Supplementary Figure 1 Comparison of overall survival between patients with a Khorana score of 1 or 2.

Supplementary Figure 2 Comparison of cancer types classified by Khorana score components, with 1 point added for other components, Cancer types 1 (base of 2-point group) **(A)**, Cancer types 2 (base of 1-point group) **(B)**, other cancer types (base of 0-point group) **(C)**.
