# Supplementary figures and images for: Association between Khorana score and prognosis in cancer patients with stage IV treated with immune checkpoint inhibitors ∼ factor analysis in Khorana score related to overall survival
Source: Front Immunol. 2025 Sep 29;16:1633398. doi: 10.3389/fimmu.2025.1633398 (PMC12515672; doi:10.3389/fimmu.2025.1633398)

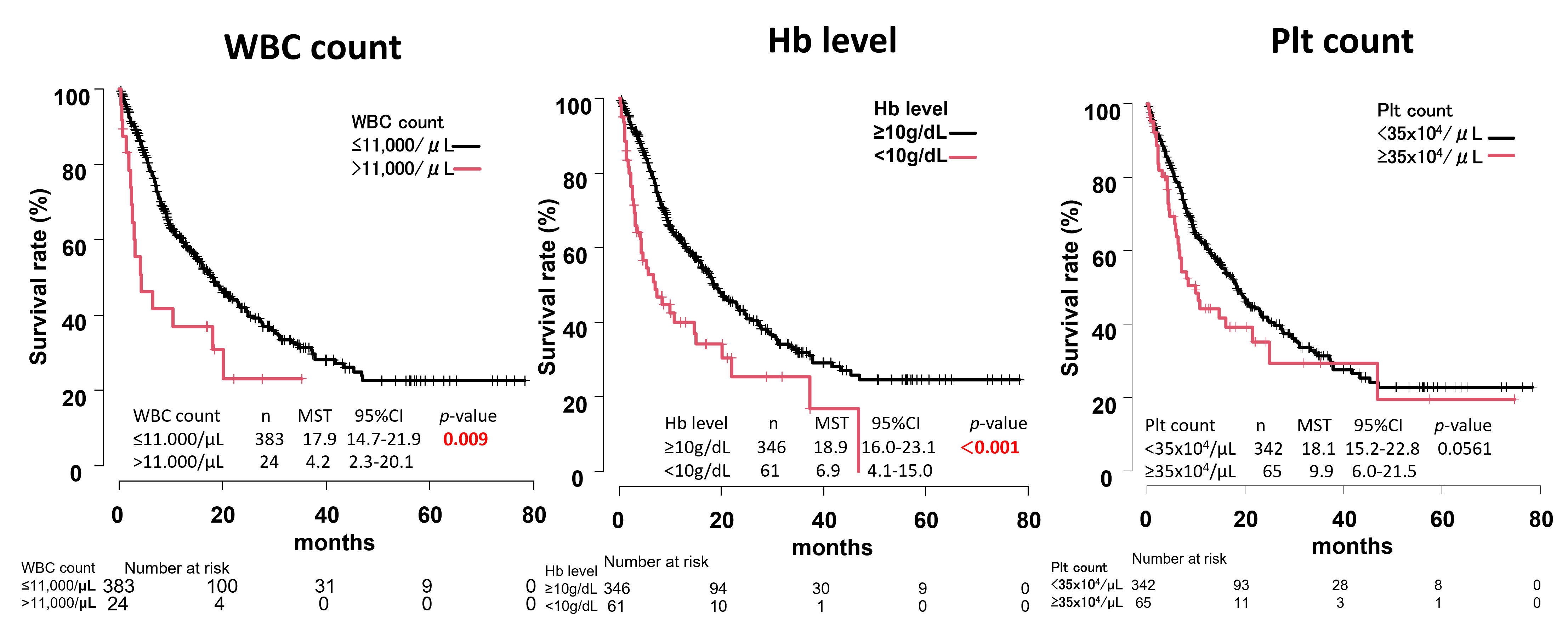

Supplement: Supplementary Figure 1 — Comparison of overall survival between patients with a Khorana score of 1 or 2. [file Image1.tif]

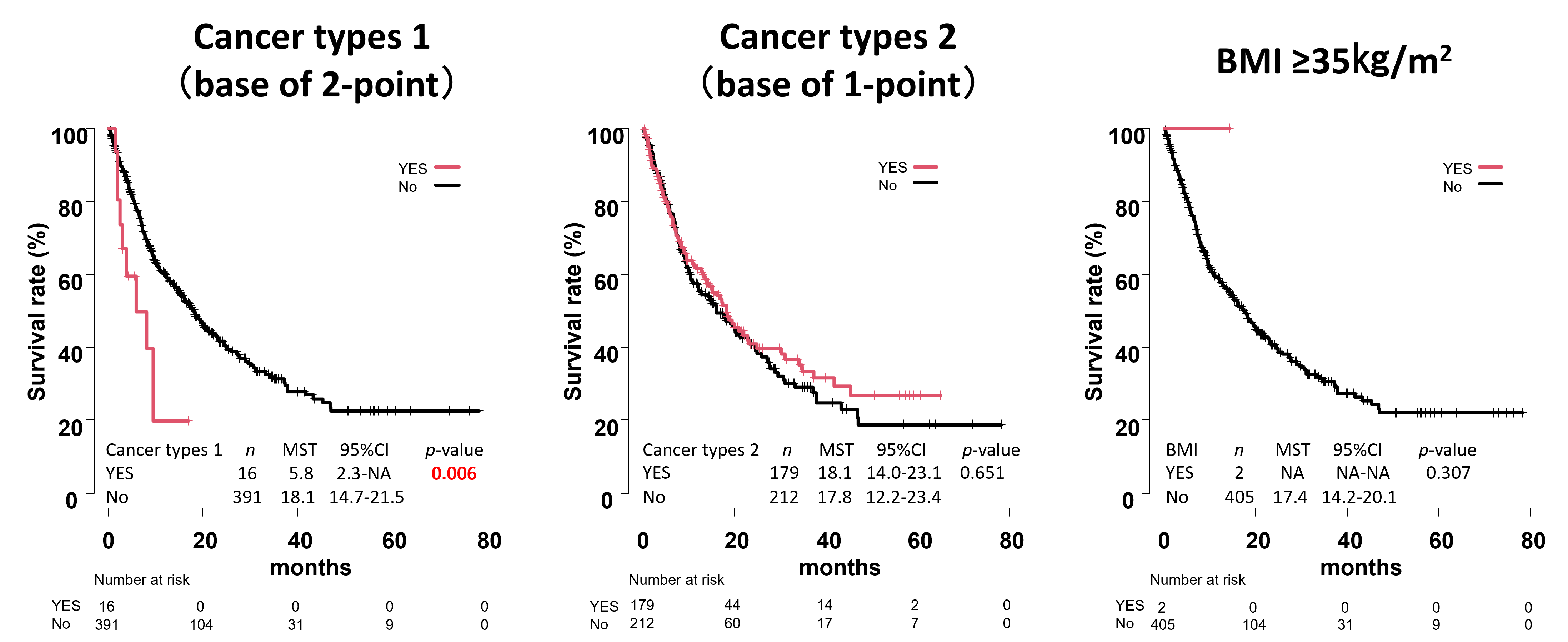

Supplement: Supplementary Figure 2 — Comparison of cancer types classified by Khorana score components, with 1 point added for other components, Cancer types 1 (base of 2-point group) (A), Cancer types 2 (base of 1-point group) (B), other cancer types (base of 0-point group) (C). [file Image2.tif]
